# A historical reflection on the discovery of human retroviruses

**DOI:** 10.1186/1742-4690-6-40

**Published:** 2009-05-01

**Authors:** Anders Vahlne

**Affiliations:** 1 Clinical Virology and Division of Clinical Microbiology, Karolinska Institutet, Karolinska University Hospital, Stockholm, Sweden

## Abstract

The discovery of HIV-1 as the cause of AIDS was one of the major scientific achievements during the last century. Here the events leading to this discovery are reviewed with particular attention to priority and actual contributions by those involved. Since I would argue that discovering HIV was dependent on the previous discovery of the first human retrovirus HTLV-I, the history of this discovery is also re-examined. The first human retroviruses (HTLV-I) was first reported by Robert C. Gallo and coworkers in 1980 and reconfirmed by Yorio Hinuma and coworkers in 1981. These discoveries were in turn dependent on the previous discovery by Gallo and coworkers in 1976 of interleukin 2 or T-cell growth factor as it was called then. HTLV-II was described by Gallo's group in 1982. A human retrovirus distinct from HTLV-I and HTLV-II in that it was shown to have the morphology of a lentivirus was in my mind described for the first time by Luc Montagnier in an oral presentation at Cold Spring Harbor in September of 1983. This virus was isolated from a patient with lymphadenopathy using the protocol previously described for HTLV by Gallo. The first peer reviewed paper by Montagnier's group of such a retrovirus, isolated from two siblings of whom one with AIDS, appeared in Lancet in April of 1984. However, the proof that a new human retrovirus (HIV-1) was the cause of AIDS was first established in four publications by Gallo's group in the May 4^th ^issue of Science in 1984.

## Background

Unfortunately the omission of the American scientist Robert C. Gallo from the 2008 Nobel Prize in Medicine or Physiology for the discovery of HIV by many has been viewed as a final scientific verdict handed down by the Nobel committee of the Karolinska Institutet on an old controversy between the Institute Pasteur and NIH and that previous settlements were for political reasons only. Also, the decision to omit Gallo has resulted in the resurrection of false allegations in the media that Gallo and coworkers at NIH had rediscovered or even stolen the French HIV isolate previously sent to them from the Pasteur Institute. Thus, it could be interpreted as if the Nobel committee finally had put right an unjust settlement previously obtained between the French and American scientific groups. There is no doubt or controversy about the fact that the French group was first to isolate this new virus. This is what the Nobel committee chose to award.

Two years ago I had the privilege to painstakingly and thoroughly go through all the literature related to the discovery of HIV. Since the motivation for the Prize by the Nobel Committee is very limited and the fact that the Committee members cannot comment on how they came to their decision, I think it is important that the medical community gets the correct historical facts about this important discovery. Therefore, I have written this article. I would say that what I present below is a fair and accurate account on the events and work that led to the discovery of a new virus as the cause of AIDS. Regarding whom should get the credit for the discovery of HIV, this review should enable the reader to come to his or her own conclusion. Mine, however, is different from that of those of my fellow faculty members that presently make up the Nobel Committee for the Nobel Prize in Physiology or Medicine. I will here show that by going through the literature it is evident that Gallo's group was not only first to show that HIV is the cause of AIDS but that the French group had not been able to discover this new virus without the active assistance of, as well as, previous work by Gallo. It will also be evident that Gallo and his associates had no reason to "steal" any French isolate. Last year this journal published another account of the 2008 Nobel Prize [1].

### Paving the way for the discovery of HIV

Isolation of a virus means infection, propagation and (usually cell free) transmission of an infectious agent in cultured cells. New viruses, for which there are no susceptible cells in culture, have lately also been detected solely by molecular techniques, e.g. hepatitis C virus by using a random-primed complementary DNA library from an infected patient (Michael Houghton) and subtypes of human papilloma viruses by using hybridization under low stringency and subsequent DNA cloning (Harald zur Hausen).

The difficulty in isolating a new virus is choosing the right cell culture and detection systems and to obtain specimens containing the virus. With a susceptible cell culture system and a detection system available, isolation of a new virus is not only possible but also rather straightforward. In the case of HIV, before the successful isolation of the first human retrovirus (human T-cell leukemia virus, now human T-cell lymphotropic virus type I; HTLV-I) by Robert C. Gallo [2], neither was at hand.

After the discovery of reverse transcriptase from animal oncogenic RNA viruses (then called oncorna viruses and now called retroviruses) a large number of publications on putative discoveries of retrovirus detections in human malignancies appeared in the early 1970-ties. However, they were all either owing to contaminations in the laboratories with animal retroviruses or the mitochondrial DNA-polymerase γ, the latter when the reports were based on reverse transcriptase activity alone. DNA-polymerase γ is a normal cellular DNA polymerase which uses RNA as a primer but not as a template. Therefore, like reverse transcriptase, the activity of DNA-polymerase γ was sensitive to a ribonuclease treatment [3-5]. This cellular enzyme was not known at the time. In 1972 Gallo's group [3] reported that stimulated normal human lymphocytes contained a ribonuclease sensitive DNA polymerase distinct from viral RNA-directed DNA polymerase, an enzyme that Gallo's group characterized further in a number of publications. The enzyme prefers Mn^2+^. Unlike DNA polymerases α and β, the preferred primer-template for DNA-polymerase γ is (dT)≅15·(A)*n *over (dT)≅15·(dA)*n*! This third cellular DNA polymerase was independently from Gallo discovered by Art Weissbach and they later named it DNA-polymerase γ[6].

From the numerous and erroneous reports on retroviruses in various human cancers, the notion of human cancer viruses became in ill repute and rather than talking of "human tumor viruses" people in science talked of "human rumor viruses". In fact, as narrated by Gallo in one of his reviews[7], when Gallo first submitted their report on HTLV-I to Journal of Virology it was rejected right away by the editor Robert Wagner "insisting that they should cease, and not continue to perpetuate the controversy, strongly implying that we all know human retroviruses do not exist".

In his quest to find a human retrovirus in lymphoma/leukemia Gallo developed sensitive and generalized techniques for the detection of reverse transcriptase to discriminate it from cellular DNA polymerases [8,9].

To isolate T cell lymphotropic viruses one needs to be able to culture T lymphocytes. Working with conditioned medium to grow lymphocytes, Gallo together with two of his post doctorial fellows Doris Morgan, Frank Ruscetti discovered T cell growth factor (TCGF) later named interleukin 2 (IL-2). Hence, the first report of IL-2/TCGF was by Robert Gallo was published in 1976 [10]. The first paper by Kendal A. Smith on IL-2/TCGF did not appear before 1978[11].

I sincerely doubt that anyone would have been looking for a retrovirus as the etiological agent for AIDS had HTLV-I not previously been isolated. I will therefore shortly recapitulate the history of the discovery of this virus.

### The discovery of the first human retrovirus

Reverse transcriptase activity was detected by Gallo's group in a T-cell line established (using IL-2) from a patients diagnosed originally with mycosis fungoides in1979. To show that this was indeed a new human retrovirus Gallo and coworkers set out to show that the same virus could be isolated from primary tissue samples of the same patient by culturing primary T-cells with IL-2; demonstrate that the virus was novel, i.e., not any of the known animal retroviruses; show it could infect human T cells *in vitro*; demonstrate specific antibodies to the virus in the serum of the patient; demonstrate that proviral DNA could be found integrated in the DNA of the cells from which the virus was isolated; and provide evidence that this was not a one-time affair by showing serological evidence of specific antibodies not only in the patient but in some others as well.

Most or all these results were obtained by the time Gallo submitted the first paper to PNAS allowing it to quickly be followed with several other reports[12]. The paper on the first isolation of HTLV-I [2] was submitted (communicated) for publication in PNAS on August 4^th ^1980 and appeared in the December issue of the same year. The second paper from Gallo and his group (especially Bernard J. Poiesz, another post doctorial fellow) on the isolation of HTLV-I now from fresh cultured cells from a patient with Sezary T-cell leukemia was submitted to Nature on May 1st 1981 and appeared in the November 19th issue of that journal[13]. In the February 19th 1982 issue of Science (submitted October 6th 1981) Gallo's group [14] reported that five of six tested ATL patients in Japan had antibodies to HTLV-I (only HTLV at the time).

On the 26th of June 1981 (six months after the Poiesz et al. paper from Gallo's group was published) Hinuma et al. submitted (communicated) a paper to PNAS showing antibodies against an antigen in a T-cell line, MT-1 from a patient with adult T-cell leukemia (ATL), in all 44 patients with ATL examined and in 32 of 40 patients with malignant T-cell lymphomas using indirect immunofluorescence[15]. The antibodies were also detected in 26% of the healthy adults examined from ATL-endemic areas but in only a few of those examined from ATL-non-endemic areas. Extra-cellular type C virus particles were detected in pelleted cells of the MT-1 T-cell line. Hinuma called this virus adult T-cell leukemia virus (ATLV). Characterization of the virus as a retrovirus was published in the March issue of PNAS, submitted (communicated) November 23rd 1981. In this paper[16] also proviral DNA was detected in fresh peripheral lymphocytes from all of five patients with ATL but not in those from healthy adults. This paper was submitted more than a month later than the Gallo paper showing antibodies to HTLV-I in Japanese ATL patients.

On July 13th of 1981 Miyoshi et al. (Hinuma last author) submitted a paper to Nature (published December 24th 1981) on the transmission of virus from MT-1 cells (female) to cord blood cells of a male infant transforming (immortalizing) the latter cells[17].

In the November 4^th ^1982 issue of Nature Gallo's group together with the Japanese colleagues Nakao, Miyoshi, Minowada, Yoshida and Ito reported that HTLV-I and ATLV was one and the same virus[18] and decided to call both viruses HTLV-I.

As is evident from the above Gallo was truly the first to isolate the first known human retrovirus and to report it. In 1982, Gallo and co-workers reported the discovery of the second human retrovirus, HTLV-II, in a patient with hairy cell leukemia. However, no malignancy or other disease has yet been clearly linked to the infection of this virus.

### The isolation of what is now called HIV-1 (will also be referred to as HIV, LAV, IDAV-1, IDAV-2, LAV-1, HTLV-III and ARV) and the demonstration of this virus as the cause of AIDS

In May of 1983 Françoise Barré-Sinoussi et al. published a paper in Science [19] describing the isolation of a putative new human retrovirus from the lymph gland of a patient suffering from persistent generalized lymphadenopathy, which is regarded as a precursor condition of AIDS. They called this new virus LAV (later LAV_BRU_) for lymphadenopathy virus and BRU from the first three letters of the patient's last name. Since this has been viewed as a seminal paper for the discovery and characterization of HIV, I will here describe this paper in detail.

Cells from a lymph node of patient B.R.U. was cultured under the conditions described by Gallo[2,13], i.e. culture medium with T-cell growth factor (TCGF or IL-2), and were stimulated with phytohemaglutinin (PHA). They also added antiserum to human α-interferon to neutralize possible endogenous interferon. (The latter is not necessary, and is not used by others.) After three days, the culture was continued in the same medium without PHA. After 15 days in culture reverse transcriptase (RT) activity was detected in the culture supernatant, using the protocol by Gallo[2,13]. Importantly, the ionic conditions were the same as for isolating HTLV-I previously described by Gallo (1 and 2; in contrast to other animal retroviruses HTLV-I has a Mg^2+^-dependent and not Mn^2+^-dependent reverse transcriptase). Virus production continued for 15 days and decreased thereafter, in parallel with the decline of lymphocyte proliferation. A standard and routine procedure in clinical virology when trying to isolate a virus is to passage the cells to fresh ones, usually when the original cells start to die, and particularly if they do not yet show any signs of being infected. Hence, to show virus transmission, cells from patient B.R.U. after three days in culture were also co-cultured with lymphocytes from a healthy donor of the Blood Transfusion Center at the Pasteur Institute. Also with these co-cultures, RT could be detected after 15 days of culture (not before) and amounts of RT remained stable for 15 to 20 days. Transmission of cell-free supernatants from the original culture of B.R.U. cells was successfully obtained using 3-day-old cultures of T lymphocytes from two umbilical cords. There is no mentioning of cytopathic effects in any of the cultures or that fresh T lymphocytes from healthy donors were added to make the virus isolation possible or to save the virus isolate except for the virus transmission experiment described above.

The virus isolate had a density of 1.16 (same as HTLV-I) in a sucrose gradient. Electron micrographs of the virus from the umbilical cord lymphocytes were reported to be of typical C-type virus, i.e. with a spherical core (same as HTLV-I). Of note, HIV is a lenti retrovirus having a conical or cylindrical core structure and does not have type C virus morphology.

Two experiments were performed to distinguish the new isolate from HTLV-I. The first was by immunofluorescense using serum from the patient as well as a goat anti HTLV-I p24 (capsid protein) and mouse monoclonal anti HTLV-I p19 (matrix protein). The two latter anti-sera, as well as two HTLV-I producing cell lines were from Robert C. Gallo as acknowledged by the French group in the paper. The sera were tested against two different cultures of normal blood lymphocytes, against the two lines of HTLV-I producing cells, and against virus producing cells from the co-culture of T lymphocytes of patient B.R.U. and the healthy donor and against infected cord blood lymphocytes. In addition cells from a lymph node from a person (patient 2) who presented with multiple adenopathies and who had been in close contact with an AIDS case was also tested. No RT activity was detected in the latter patient's cultured lymphocytes. The anti-HTLV-I sera from Gallo (anti p19 and p24) reacted with the HTLV-I producing cell lines only. Serum from patient B.R.U. reacted with 90–100% of the HTLV-I producing cell lines and with 90–100% of the co-cultured cells from B.R.U and the healthy donor, as well as, the cells from patient 2. The B.R.U. serum reacted with only 0.5 to 2% of the infected umbilical cord lymphocytes. It is noteworthy that B.R.U.'s serum reacted with 90–100% of the co-cultured cells from B.R.U and the healthy donor since we know that only the CD4 positive cells should be infected. The B.R.U.'s serum also reacted with 90–100% of the HTLV-I producing cells! If this were to be due to a possible double infection with HIV and HTLV-I again only CD4 positive cells should be positive. More likely something unrelated to either HIV or HTLV-I was detected by the B.R.U. serum, in my opinion most probably mycoplasma, a common contaminant in cell culture. The 0.5 to 2% positive infected umbilical cord lymphocytes may indicate retrovirus-infected cells. However, the lack of reactivity with the p19 and p24 sera with these cells is not a proof that the B.R.U. virus was not HTLV-I. The few percentages of possibly positive cells could simply have been missed with the specific antibodies but detected with the patient's sera containing antibodies to all viral proteins. The paper does not present any photos of the fluorescent cells.

The other experiment performed to distinguish the new virus from HTLV-I was immunoprecipitation of lysates of infected cord lymphocytes, as well as, virus released from the infected cells with the same sera used for immunofluorescense and in addition serum from patient 2. Serum from B.R.U. and patient 2 (whose lymphocytes were RT negative) precipitated a protein of the apparent size of 25,000 from extracts of the infected cord lymphocytes and from the supernatants of these cells. Serum from a healthy donor did not precipitate this protein, nor did the anti HTLV-I p19 or p24 sera. The sera from B.R.U. and patient 2 did not precipitate the p25 protein from an extract of one of the HTLV-I producing cell lines. However, neither did the goat anti HTLV-I serum from what I can determine from the figure presented! Thus, there is no positive control indicating that they indeed had an HTLV antigen to precipitate. If the goat antiserum indeed precipitated a p24 protein from the HTLV-I producing cell extract, the band of p24 precipitated was extremely week, indicating that the serum was not very good at precipitating HTLV-I p24, at least not in the hands of Barré-Sinoussi and coworkers. The HTLV-I producing cells were all infected as opposed to only 0.5 to 2% of the cord blood cells. So, if the cord blood cells were infected with HTLV-I and not a new virus, the goat antiserum would still have had a hard time precipitating any protein! An appropriate control would have been a serum from a HTLV-I infected individual. The size of the protein they precipitated is in fact 24,000, the same as that of HTLV-I. The core protein of both HIV and HTLV-I is 24,000.

In reality, in my view there is no evidence whatsoever in this paper that a new human retrovirus has been isolated! With the data presented, the virus they isolated could well have been HTLV-I or in particular HTLV-II. The paper was obviously written in haste, as acknowledged by Montagnier[20], and contains numerous errors and omissions in the figures legends.

After having a first manuscript being rejected by Nature, Gallo suggested to Montagnier to send it to Science and even strongly endorsed the paper to the journal (the book of Nikolas Kontaratos shows a facsimile of Gallo's letter to the editor of Science[21]).

A more thorough description of the French isolate LAV from patient BRU and two new retrovirus isolates from two patients with AIDS was given at a Cold Spring Harbor meeting held in September of 1983. A proceedings, however not peer reviewed, from the meeting was published not until September of 1984[22]. The oral presentation by Luc Montagnier at this meeting is to my mind the first report on a new third human retrovirus, in that electron micrographs on the isolate LAV from patient BRU clearly showed virus with conical cores. A selective tropism of LAV to CD4 positive T-cells (as is the case for HTLV-I) was also demonstrated.

The first publication in a peer reviewed journal indicating the isolation of a new retrovirus, distinct from HTLV-I and HTLV-II, isolated from two siblings with hemophilia B of whom one with AIDS, appeared in Lancet in April of 1984 and was written by the French group[23]. Again the immunological and molecular characterization of the isolated virus does not convincingly separate the isolated virus from HTLV-I. However, an electron micrograph clearly depicts a virus with a lenti retrovirus type morphology having a cylindrical or conical core, distinctly different from the larger spherical core of HTLV-I, and HTLV-II. The paper, however, fails to conclusively link the new virus as the causative agent of AIDS.

In conclusion, by April of 1984 the Pasteur group headed by Luc Montagnier had reported on a new human T-lymphotropic retrovirus distinct from HTLV-I and HTLV-II as judged by morphology and which was present in a few patients with AIDS and lymphadenopathy, as well as, in people at risk of acquiring AIDS. The virus infected CD4-positive T-lymphocytes, the very cells affected in AIDS. Although clearly associated with AIDS, they had not yet shown that the new virus was an etiological agent, and the only one at that, of this new disease.

On May 4^th ^of 1984 four papers by Robert C. Gallo's group were published in Science describing a new human retrovirus virus as the probable cause of AIDS. All four papers were submitted the 30^th ^of March 1984. One paper[24] describes the isolation of the new virus from cultured lymphocytes obtained from 48 different individuals. The culturing technique was what had previously been described by Gallo and which Montagnier's group also used. The new cytopathic (large multinucleated cells) virus isolates were collectively designated HTLV-III and was characterized by having a Mg^2+^-dependent reverse transcriptase, being transmittable by co-cultivation of T cells with irradiated donor cells or with cell free fluids, having distinct morphology by electron microscopy, and by expressing specific viral antigens (indirect immune fluorescence) using a serum obtained from a patient with pre-AIDS (described in an adjoining papers 25 and 26; this serum did not react with cells infected with HTLV-I or HTLV-II), as well as, antisera prepared against purified, whole disrupted HTLV-III. The 48 HTLV-III isolates were obtained from 18 of 21 tested patients with unexplained lymphadenopathy and leukopenia, with an inverted T4/T8 lymphocyte ratio (designated pre-AIDS), 3 of 4 clinically normal mothers of juvenile AIDS patients, 3 of 8 juvenile AIDS patients, 13 of 43 adult AIDS patients with Kaposis sarcoma, 10 of 21 adults AIDS patients with opportunistic infections, and 1 of 22 clinically normal homosexual donors. Importantly, this homosexual donor, from whom HTLV-III was isolated, developed AIDS six month after the virus isolations were performed. This means that these isolations were performed not later than September of 1983. HTLV-III could not be isolated from any of 115 clinically normal heterosexual donors.

In a second accompanying paper [25] antibody reactivity to HTLV-III antigens in patients with pre-AIDS and AIDS was determined by an enzyme-linked immunosorbent assay (ELISA) as well as a Western electrophoretic blotting technique using a lysate of sucrose gradient purified HTLV-III from a cell line continuously producing HTLV-III. [26] as antigen. The number of sera that gave positive scores in the ELISA were: 43 of 49 (88%) of patients with AIDS (two of whom had developed AIDS after blood transfusion), 11 of 14 patients with pre-AIDS, 3 of 5 intravenous drug users (of which one positive was also homosexual), 6 of 17 homosexual men. It is noted in the paper that these homosexual men had been seeking medical assistance; they probably were not representative of the homosexual population. Out of 186 controls only one scored positive in the ELISA (1 of the 164 normal subjects). The controls also included 3 patients with hepatitis B virus infection, 1 with rheumatoid arthritis, 6 with systemic lupus erythematosus, 4 with acute mononucleosis, and 8 patients with lymphatic leukemias. Of the latter some were positive for HTLV-I. None of these 22 control patients scored positive in the ELISA or Western blot. Of note, in Western blot the antigen most prominently and commonly detected among all of the sera from AIDS patients had a molecular weight of 41,000 (now designated gp41). It was presumed that this is a virus envelope protein (which later turned out to be correct). Others, including myself, have later confirmed that gp41 is extremely reactive in ELISA of sera from HIV infected individuals. In fact we have found that an ELISA having as only antigen a peptide with the amino acids GKLICT, representing an epitope of gp41, reacts positively with the majority of sera from HIV infected individuals.

The French group did not detect gp41 in their immune precipitation studies using purified LAV. Their inability to detect this protein in their ELISA or immune precipitation experiments is probably the main reason that their positive scores with AIDS and pre-AIDS sera were so low. HIV is an enveloped virus and hence fragile. Most certainly they had lost the virus envelope in their purification of the virus.

Taken together, these two papers from Gallo's group for the first time convincingly demonstrated that AIDS was caused by a new human retrovirus distinct from HTLV-I and HTLV-II. It also provided with a blood test (ELISA) by which blood donors could be screened and a confirmation assay (Western blot) for those who tested positive in the ELISA. The authors speculate that the virus they found could well be the same virus that was previously detected by the French group, but direct comparisons had not yet been performed.

A third paper. [26] describes the establishment of cell lines continuously producing HTLV-III. A total of 51 single cell clones (designated H1 to H51) were obtained from a neoplastic aneuploid T-cell line (HUT-78). The clones were tested for susceptibility to concentrates of HTLV-III. All clones were susceptible and permissive for the virus, but virus yields and cell proliferation varied considerably. The best clones (H4 and H9) were used for the long-term propagation of HTLV-III from patients with AIDS and pre-AIDS. Five different isolates using the H4 or H9 clones are presented. Four were obtained by co-cultivating the patients T-cells with the H4 cells and one by infecting H9 cells with a cell free concentrated culture fluid harvested from T-cell cultures from a patient (W.T.) who had lymphadenopathy. One was from an AIDS patient from Haiti (R.F.) and four were from the US. In the paper they also report that some of the 48 isolates described in the accompanying paper[24] also could be propagated in the H4 and H9 clones. The importance of this paper is that for the first time it was shown that one could propagate HIV in large quantities as a source for antigen in a blood test, as well as, for in depth characterization of the virus. It was this paper, and the patent which was based upon it, that later caused the controversy between the NIH and the Pasteur Institute. It turned out that the HTLV-III producing H9 clone selected for the blood test was in fact a pick-up of a French HIV isolate sent to Gallo in September of 1983. This will be discussed later.

The fourth of the Gallo Science papers [27] describes a first attempt to serologically characterize HTLV-III using Western blot and sera from AIDS and pre-AIDS patients. The paper describes for the first time a virus protein of approximately 130,000 (in fact it is 120,000 and now designated gp120). Also a protein of 55,000 (p55) is described and correctly concluded to be a precursor protein for the capsid protein p24.

Lastly, a photomontage of electron micrographs of HTLV-I, HTLV-II, and HTLV-III with budding virus particles, immature virus particles and mature virus particles is shown. Although the budding and immature virus particles are very similar for all three viruses, the mature HTLV-III viruses are distinctive from those of HTLV-I and HTLV-II.

Three more papers on antibody reactivity to LAV/HTLV-III in patients with AIDS or pre-AIDS were published in the summer of 1984. June 9^th^, Montagnier's group[28] published an ELISA based on purified virus particles. The presented results were: 18/48 (37.5%) of AIDS patients, 38/51 (74.5%) of pre-AIDS patients and 8/44 (18%) of homosexual men without pre-AIDS, but only one of 100 unselected blood donors were positive. In a note added in proof they claim that by modifying their assay now 75% of AIDS patients and 90% of pre-AIDS patients scored positive. In the Lancet issue of June 30^th ^Gallo's group[29] publish their second report (the first being the one in Science above) on ELISA and Western blot confirmatory assay in a double-blind seroepidemiological study. The composite result of the two assays gave: 34 of 34 AIDS patients were positive (100%), 16 of 19 (84%) of lymphadenopathy (pre-AIDS), 3 of 14 (21%) at risk for AIDS, and none of 14 controls were positive. Lastly, Kalyanaraman et al. [30] published a paper the 20^th ^of July in Science submitted May 4^th ^1984. This paper was from Donald Francis group at the Center for Disease Control, Atlanta, in collaboration with Montagnier's group at Pasteur. The assay they used was based on immuno-precipitation. The positive scores were: 51 of 125 (41%) of AIDS patients; 81 of 113 pre-AIDS patients, 0 of CDC workers, and 0 of 189 random blood donors. Of 100 blood samples collected in 1978 from homosexual men in San Francisco, only one was positive as opposed to 12 of 50 such sera collected in 1984.

In the July 6^th ^issue of Science (submitted April 6^th ^1984), Donald Francis' group in collaboration with Montagnier's group reported on the isolation of a retrovirus from a blood donor-recipient pair with AIDS [31]. In an elegant experiment, using a competition radioimmunoassay they clearly show that the viruses they isolated were closely related to LAV but not to HTLV-I or HTLV-II. This is the first paper to show transmission of HIV-1 from one patient to another. This is also the first time, beside the electron microscopic pictures of LAV, Montagnier convincingly shows that LAV is antigenic distinct from HTLV-I.

On August 24^th ^1984 Jay Levy in San Francisco[32] published a paper in Science (submitted May 31^st^) reporting that using the Gallo protocol they had isolated a retrovirus with lenti retrovirus type morphology designated ARV for AIDS associated retrovirus in 22 of 45 patients with AIDS. Positive virus cultures were also received from 5 of 10 patients with lymphadenopathy (pre-AIDS), 3 of 14 male sex partners of AIDS patients, 2 of 9 clinical healthy homosexual men, and 1 of 23 clinically healthy heterosexual men. When tested in immune fluorescence with slides containing acetone fixed cells infected with ARV, HTLV-I or LAV, 78/86 (91%) of AIDS patient's sera were positive to ARV infected cells, 22 of 40 (55%) to LAV, and 8 of 60 (13%) to HTLV-I. None of 56 controls reacted to any of the virus-infected cells. The fixed ARV infected cells were from a cell line (HUT-78) successfully established to continuously produce ARV.

### The LAV/HTLV-IIIB contamination story and the patent feud between the Pasteur Institute and NIH

Right before the first public announcement by Barré-Sinoussi at a conference at Cold Spring Harbour in May of 1983 of the Pasteur group's findings, the Pasteur Institute filed a patent for the virus they had isolated. Before going public with the four Science papers, the NIH filed for a patent for the blood test described in one of the papers to be published in May of 1984. The United States patent office quickly allowed the American patent, shortly to be followed by allowances from European patent offices, and a number of American companies started to produce and sell blood tests. The approval of the Pasteur patent was delayed, principally because the French had not reduced their patent to practice, i.e. showed that they had a working blood test in the patent application. This led to a patent feud between the NIH and the Pasteur Institute starting in August of 1985. To solve this feud, the governments of both countries had to become involved. The patent fight came to an end on March 31^st^, 1987, when President Ronald Reagan and French Prime Minister Jacques Chirac signed an agreement to settle the arguments. The financial outcome of the agreement, however, did not turn out to the satisfaction of the French, and when it became clear that the US patented blood test was based on a laboratory contamination of a French virus the deal was re-negotiated in 1994. It should be stated right away that neither of the scientists at the time stood to gain from respective patent.

The ground for the feud was the following. Montagnier sent his first isolate LAV_BRU _to Gallo in July of 1983. In May of 1984 Gallo's coworker Sarngadharan brings one of Gallo's five HIV strains (HTLV-III_B_) that grew well in a continuous cell line to Montagniers laboratory in Paris. In July of 1984 Montagnier sends Gallo a second sample of LAV_BRU _since Gallo had complained that the first didn't grew well at NIH. Gallo then found and reported[33] that HIV was extremely variable; every isolated strain was different from the other also when obtained from the same individual but at different times. However, the two strains LAV_BRU _(received in July of 1984) and HTLV-III_B _isolated on either side of the Atlantic Ocean where strikingly similar. Gallo's reaction to this was that Montagnier must have contaminated his cultures with the American isolate, i.e. that the Pasteur group had had a so-called "pick-up" of HTLV-III_B _into his poorly replicating LAV_BRU_. Gallo gave Montagnier a call, but the latter denied that this could have happened in his laboratory. Since the LAV_BRU _obtained by other laboratories, before HTLV-III_B _had been introduced to the Pasteur laboratory, had a genome more or less identical to the French isolate, it was concluded that the contamination must have happened in Gallo's laboratory. Gallo found this very strange, since LAV_BRU _replicated very poorly and could not be transferred to a continuously producing cell line like they had achieved with HTLV-III_B_. On Sunday, November 19^th^, 1989, the Chicago Tribune published a 16 pages account by journalist John Crewdson of the discovery of HIV. The article concludes that HTLV-III_B _is LAV_BRU_. Crewdson implied in not so subtle words that Gallo had stolen the virus from the French. This started three separate investigations for scientific misconduct by Popovic and Gallo that didn't end until November of 1993.

The explanation came in 1992. Sequencing the original isolate LAV_BRU _received in NIH in 1983, Gallo found that it was different from the LAV_BRU _received in July of 1984. The original LAV_BRU _was as expected of the slowly replicating CCR5 co-receptor using genotype whereas LAV_BRU _from 1984 was of the rapidly replicating CXCR4 using genotype. In fact the 1984 LAV_BRU _was identical to LAV_LAI_. Thus, the contamination had originally occurred in the Pasteur laboratory. According to Montagnier at least six other laboratories received the LAI sample (under the name BRU) from his group and experienced the same contamination. [34]. Montagnier speculates that this was due to *Mycoplasma pirum *contamination of the cultures infected with LAV_LAI_. In his review "A history of HIV discovery" in Science. [34] Montagnier writes: "This physical association makes a fraction of the LAI virus highly infectious, and, in fact, this fraction can be neutralized with antibodies against *M. pirum*. As mycoplasmas are common contaminents of cultured cells, an infectious pseudotype virus (LAI associated with *M. pirum*) may have caused several contaminations between 1983 and 1984 in different laboratories".

In Gallo's laboratory LAV_BRU _had contaminated a pool of viruses from different AIDS patients. Pooling viruses was the idea of Mika Popovic in order to get the "survival of the fittest" to grow out in the continuous cell line H9, a subclone of the HUT-78 cell line. The virus that grew best was named III_B _because it was from the B-pool of two pools in culture.

Many probably thought that the finding that a contamination had taken place already in the Pasteur laboratory was "convenient" for Gallo. However, to "steal" the French virus Gallo must have had a motive. One would have been that they couldn't culture any virus from AIDS patients and were becoming desperate. However, Gallo had already recognized that the French group was first to isolate a new retrovirus from AIDS patients. More so he endorsed the publication of the first Pasteur paper, which (although in my opinion erroneously) claimed so. Secondly, Gallo's group already had 48 (sic!) isolates many growing short term in their laboratory of which five were growing in continuous cell lines[24,26]. Gallo's misfortune was that he decided to choose the III_B _for the blood test and for further characterization of the virus. Had he chosen a Haitian strain, which he also had growing long term in the laboratory at the time, we now know that he would have chosen a virus, which was as much of a prototype strain for HIV-1.

It has been questioned whether Gallo indeed had all those isolates. Considering that Gallo published this and that he and his laboratory was scrutinized for almost five years by three different investigations, had Gallo not had those isolates he would for sure have been found guilty of scientific misconduct and expelled from the NIH. Going through 13 foot high pile of Gallo's lab records including laboratory note-books, some 10,000 man hours of interviews with laboratory personal and other witnesses, all the Office of Research Integrity (ORI; a non-scientist office of government consisting of lawyers and administrators) could come up with in criticism was for Mika Popovic that he wrote "ND" in two occasions in one published table (in paper 25), and found him therefore guilty of scientific misconduct. The table legend didn't define "ND" and it was the ORI's understanding that "ND" meant, "not done" and that Popovic indeed had performed the experiment. However, Popovic insisted that by "ND" in his notebook he meant, "not determinable". The paper was written while Popovic was on holiday back in Czechoslovakia. Following Popovic's appeal to the Research Integrity Adjudications Panel the decision of ORI's was reversed. Gallo was temporarily criticized by the ORI for having written a sentence in the discussion of the same paper that LAV_BRU _had not yet been growing well enough to make possible comparisons with III_B _when in fact a technician of Popovic's had done so. These charges were later dropped by the ORI. In its decision on the Popovic case the Departmental Appeals Board's Research Integrity Adjudications Panel writes: "One might anticipate that from all this evidence, after all the sound and fury, there would be at last a residue of palpable wrongdoing. This is not the case". It is safe to say that whatever Gallo claimed he had, he had.

## Conclusion

There is no doubt that Luc Montagnier's group at the Pasteur Institute in Paris was the first to isolate the causative agent of AIDS. Montagnier, however, got the idea to try to isolate a retrovirus indirectly from Robert Gallo and Myron Essex. The protocols he used for virus isolation and RT detection were developed by Robert Gallo and the reagents he used to discriminate the new virus from HTLV-I and HTLV-II were obtained from Robert Gallo. Moreover, it is well known that Francoise Barré-Sinoussi had spent time in Robert Gallo's laboratory to learn to culture lymphocytes. Robert Gallo was the first to convincingly show that the new human retrovirus (HIV) was the causative agent of AIDS, and the only one at that. He also was also the first to provide a blood test to screen blood donors for HIV infection. The rapid implementation of the latter in the US and Europe probably saved hundreds of thousands of lives.
